# Dietary Derived Micronutrients Modulate Immune Responses Through Innate Lymphoid Cells

**DOI:** 10.3389/fimmu.2021.670632

**Published:** 2021-04-29

**Authors:** Zhengzheng Shi, Hiroshi Ohno, Naoko Satoh-Takayama

**Affiliations:** ^1^ Laboratory for Intestinal Ecosystem, RIKEN Center for Integrative Medical Sciences, Yokohama, Japan; ^2^ Laboratory for Immune Regulation, Graduate School of Medical and Pharmaceutical Sciences, Chiba University, Chiba, Japan; ^3^ Immunobiology Laboratory, Graduate School of Medical Life Science, Yokohama City University, Yokohama, Japan; ^4^ Intestinal Microbiota Project, Kanagawa Institute of Industrial Science and Technology, Kawasaki, Japan

**Keywords:** micronutrients, innate lymphoid cells, AhR ligands, vitamin A and D, mucosal protection, homeostasis regulation

## Abstract

Innate lymphoid cells (ILCs) are a group of innate immune cells that possess overlapping features with T cells, although they lack antigen-specific receptors. ILCs consist of five subsets-ILC1, ILC2, ILC3, lymphoid tissue inducer (LTi-like) cells, and natural killer (NK) cells. They have significant functions in mediating various immune responses, protecting mucosal barrier integrity and maintaining tissue homeostasis in the lung, skin, intestines, and liver. ILCs react immediately to signals from internal and external sources. Emerging evidence has revealed that dietary micronutrients, such as various vitamins and minerals can significantly modulate immune responses through ILCs and subsequently affect human health. It has been demonstrated that micronutrients control the development and proliferation of different types of ILCs. They are also potent immunoregulators in several autoimmune diseases and play vital roles in resolving local inflammation. Here, we summarize the interplay between several essential micronutrients and ILCs to maintain epithelial barrier functions in various mucosal tissues and discuss their limitations and potentials for promoting human health.

## Introduction

Innate lymphoid cells (ILCs) are a family of innate immune cells that possess overlapping characteristics with T cells. ILCs exhibit properties of CD4^+^ helper T (Th) cells and CD8^+^ cytotoxic T (Tc) cells, although they lack the antigen-specific receptors of adaptive immune cells. ILCs can be divided in several subgroups, defined mainly by the intrinsic transcription factors expressed and the cytokines produced by each subgroup. ILCs were initially categorized into three major subgroups: ILC1, ILC2, and ILC3. Recently, the nomenclature of ILCs has been updated based on a more in-depth understanding of the unique developmental pathways they follow; the latest nomenclature reclassified ILCs into five subgroups: ILC1, ILC2, ILC3, lymphoid tissue inducer (LTi-like) cells, and natural killer (NK) cells (1, 2). ILCs commonly express CD127, the IL-7 receptor α chain (IL-7α) which supports their survival and proliferation. ILC1s require T-bet to function and produce interferon (IFN)-*γ* (3). ILC2s are characterized by a high expression level of the GATA3 transcription factor and the capacity to produce large amounts of type 2 cytokines, IL-4, IL-5, and IL-13 (2, 4, 5). ILC3s rely on the transcription factor retinoic acid-related orphan receptor *γ* isoform t (ROR*γ*t) to differentiate and survive (6). There are two kinds of ILC3s. One consists of cells that expresses the surface marker NKp46, termed natural cytotoxicity receptor (NCR)^+^ ILC3s, and are significant sources of interleukin 22 (IL-22). The other subgroup is LTi-like cells, which express the C-C motif chemokine receptor 6 (CCR6) but lack NKp46 expression (6, 7). LTi cells require ROR*γ*t for their development, act to generate the secondary lymph nodes and Peyer’s patches during fetal development and mainly produce the cytokine IL-17 (8, 9). NK cells are regulated by both T-bet and Eomesodermin (Eomes) and they are potent cytotoxic cells found within tissues or circulating in the blood (10).

**Figure 1:**
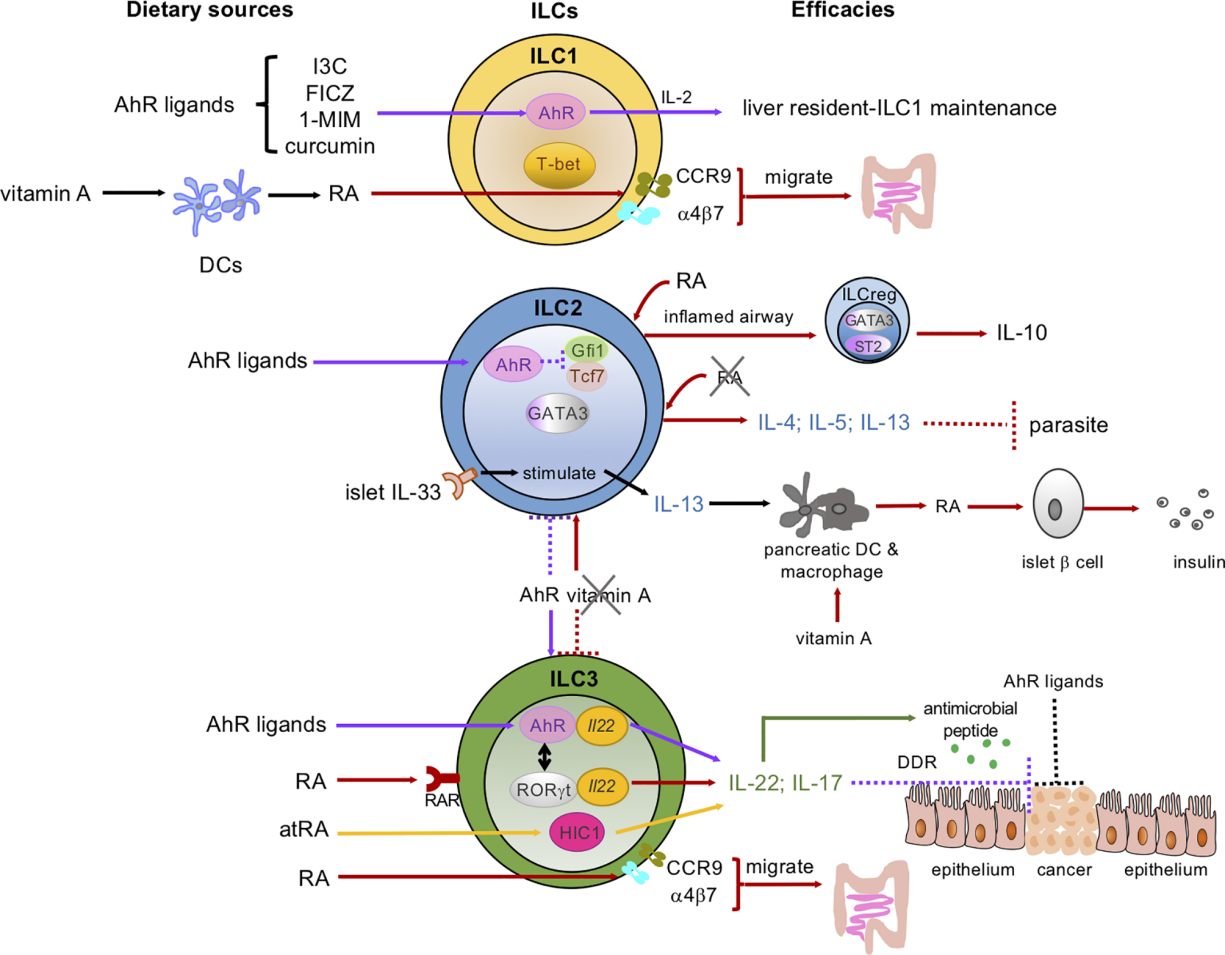
Dietary micronutrients have various and sophisticated programs to mediate the development, proliferation, and functions of ILCs. Dietary sources of micronutrients: AhR ligands, vitamin A and its metabolites, retinoic acid (RA) and *all-trans* RA (atRA). These micronutrients interact with ILC subsets: ILC1s, ILC2s, ILC3s in the intestine, pancreas, liver, and lung. AhR signaling (indicated as purple arrows): AhR ligands maintain the liver-resident ILC1s/NK cells. AhR is highly expressed by gut ILC2s and inhibits *Gfi1* and *Tcf7* gene expression, while sustaining ILC3s to control the ILC2-ILC3 balance (shown in the purple dotted line and the purple arrow between the ILC2 and ILC3). In ILC3s, both AhR and ROR*γ*t bind to the *Il22* locus (yellow ovals in the ILC3) and promote IL-22 and antimicrobial peptide secretion (green arrows and dots). AhR ligands bind to AhR and enhance ILC3s to secrete IL-22 and prevent intestinal epithelial cells (IECs) from becoming transformed via the DNA damage response (DDR) (as shown in the purple dotted line). AhR ligands may also directly act on IECs to prevent malignant transformation. Vitamin A/RA signaling (indicated as red arrows and atRA as yellow arrows): RA induces the expression of gut-homing receptors CCR9 and α4β7 on both ILC1s and ILC3s and guides them to migrate to the gut. RA can also convert some airway ILC2s to regulatory ILCs (ILCreg), which express IL-10, in the inflamed tissues in the presence of the cytokines IL-33 and IL-2. In pancreatic-islets, IL-33 activates local ILC2s and the IL-33-ILC2 axis imprints RA-producing activity in DCs or macrophages and promotes insulin secretion by β cells. RA and atRA promote IL-22-producing NCR^+^ ILC3s and IL-17-producing ILC3s. A vitamin A-deprived diet enhances ILC2 functions and associated cytokines, including IL-4, IL-5, and IL-13 (indicated in red arrow between the ILC2 and ILC3), and showed resistance to parasitic infection. Meanwhile, the vitamin A-deprived diet suppresses ILC3s and their cytokines (red-dotted line between the ILC2 and ILC3). Solid lines represent the enhanced signaling pathways, while the dotted lines represent the suppressed signaling pathways.

ILCs have significant biomedical functions in tissue homeostasis, mediating innate immunity and communicating with adaptive immunity, and are involved in the pathogenesis of multiple autoimmune diseases (7, 11–13). Considerable emerging evidence has established that tissue-resident ILCs sense and promptly respond to perturbations in internal physiological responses to maintain the homeostasis of mucosal tissues. In addition, they react to an even more comprehensive range of challenges from external sources (e.g., dietary constituents, microbes, and pollutants). Indeed, our daily diet, which contains plenty of nutrients, energy sources and toxins, may vitally and vigorously affect our innate immunity by changing the immune cell cycle and cell fate, and can impact autoimmune dysfunctions and cancer (14, 15). For example, the western-style high calorie diet could induce long-lasting transcriptional and epigenetic reprogramming of monocytes and enhanced proliferation of myeloid progenitor cells due to diet-associated systematic inflammation (16). Micronutrients, including vitamins and minerals, are essential for the immune system to function efficiently, despite their low quantities in the body. Micronutrient deficiency leads to imbalanced host defense and increases the infection risk and immune dysregulation throughout different life courses (17). Along with the increasing insight into ILCs that we have gained in the last decade, the linkage between micronutrients and ILCs has also become a timely topic. In fact, recent work has highlighted the ability of ILC3s to respond to various dietary stimuli and the ILC3s transcriptional program could be precisely modified by several dietary metabolites (7). In this review, we summarize the current understanding of the communication between several essential micronutrients, mainly Aryl hydrocarbon receptor (AhR) ligands, vitamin A and D, and ILCs to defend the mucosal tissue epithelial barrier and discuss their limitations and potentials for promoting human health (**Figure 1** and **Table 1**).

**Table 1:** Micronutrients and associated signaling pathways that affect innate lymphoid cells (ILCs).

Dietary Source / signaling pathway	Targeted ILCs	Descriptions and other impacts	Refs
AhR ligands / AhR signaling	ILC3s	Anti-bacterial infection; formation of cryptopatches and ILFs	(18)
	ILC3s	IL-22 secretion; anti-colon cancer	(19)
	ILC3s	IL-22 secretion; balance between cytochrome P450 1 (CYP1) activation and its feedback	(20)
	ILC3s	AhR-RORγt interaction for IL-22 secretion; anti-enteric infections	(21)
	ILC3s	IL-22 secretion; formation of cryptopatches and ILFs in postnatal phase; Notch dependent and independent regulation	(22)
	ILC2s & ILC3s	Inhibit ILC2; sustain ILC3 to control ILC2-ILC3 balance; Enhance ILC2 immunity against helminth infection in AhR KO mice	(23)
	ILC1s / NK cells	Loss of memory-type immunity by lack of AhR expression	(24)
Vitamin A / RA signaling	ILC2s & ILC3s	Enhance ILC2 and type 2-cytokine production, IL-4, IL-5 and IL-13 in Vitamin A-insufficient (VAI) diet ILC2 induction upon helminth infection under VAl diet; Reduce IL-22-producing ILC3 upon VAl diet	(25)
	LTi cells	Modulate embryonic lymphoid organogenesis; control the efficacy of offspring immunity	(26)
	ILC3s	Reduce IL-22-producing ILC3s via HIC1; anti-bacterial infections	(27)
	ILC1s & ILC3s	CCR9 and α4β7 gut homing receptor activation; gut homing functions	(28)
	ILC2s	Upregulation of fatty acid usage in the absence of RA	(29)
	ILC2s	Induce ILCreg from ILC2s in human inflamed airway *in vitro*	(30)
	ILC2s	IL-33 activated pancreatic-islet ILC2s and imprint RA-producing functions to DCs and macrophages; enhance insulin secretion	(31)
	NK cells	Infiltration of NK cells into tumor region; promote NK cell cytotoxicity	(32, 33)
Vitamin D / VDR signaling	ILC3s	IL-22 secretion; enhance host defense against experimental colitis	(34, 35)
	ILC3s	Upregulation of VDR in human NKp44^+^ILC3s; downregulation of IL-23R pathway and cytokines	(36)
	ILC3s	Increase IL-22-producing ILC3s by depletion of VDR; Induction of dysbiosis with less susceptible to *C. rodentium* by VDR depletion	(37)

## Dietary AhR Ligands Serve as Key Factors in Innate Immunity

The AhR is a ligand-dependent transcription factor that plays a vital role in cell cycle and cell fate, maintenance of barrier functions and regulating immune responses (15). AhR ligands come from both external and internal sources. Diverse dietary components have been considered as fundamental exogenous sources of AhR ligands. For example, vegetables of the *Brassica genus*, also called cruciferous vegetables, such as cabbage, cauliflower, broccoli, brussels sprouts, etc., the herb turmeric, green tea, and citrus fruits contain various AhR-binding compounds. After consumption, the body converts the *Brassica genus* vegetables-derived components to secondary metabolites such as indole-3-carbinol (I3C) and its derivative indolo [3,2-b] carbazole (ICZ), which possesses a significantly high affinity for the AhR (38). Of note, AhR was originally discovered as the high-affinity receptor for the environmental pollutant 2,3,7,8-tetrachlorodibenzop-dioxin (TCDD), or dioxin. Dioxin is a potent promoter of carcinoma in rodent models (39). Several metabolites synthesized by host commensal microorganisms and from certain amino acids are the origin of the endogenous AhR ligands (14, 38). One of the most extensively studied endogenous AhR ligands, 6-formylindolo [3,2-b] carbazole (FICZ), is a tryptophan-derived AhR ligand that has been shown to bind to the AhR with the highest affinity (40). Indeed, FICZ upregulates the expression of stem cell factor receptor (c-Kit) and IL-22 in the human immune cells (41), and FICZ-stimulated AhR signaling has been suggested to be a two-edged sword in tumorigenesis (42).

AhR is an ancient gene that is ubiquitously expressed by vertebrate cells and, more recently, it has been recognized as a significant regulator of immune cells. Numerous studies have established that T cells, such as Th17 cells in both peripheral blood and spinal cord (43), and TCRαβ/CD8αα and TCR*γ*δ intraepithelial lymphocytes (IELs) in the intestine and epidermis, require AhR for their persistence (44). In addition, compelling evidence indicates that AhR signaling plays a vital role in innate immunity. Initially, an *in vitro* study showed that AhR directs the transcriptional activity of peritoneal macrophages stimulated by LPS-induced proinflammatory responses (45). Subsequently, recent findings have shown that ILC3s, including both LTi-like ILC3s and NCR^+^ ILC3s, require AhR for their development and maintenance, and facilitate IL-22 production in small and large intestines to sustain microenvironmental homeostasis (18, 21, 22). ROR*γ*t can also interact with the AhR to enhance *Il22* gene binding and promote IL-22 secretion (21). In addition, impaired ILC3s in the postnatal phase hamper the organogenesis of secondary lymphoid tissues including cryptopatches and isolated lymphoid follicles (ILFs). The latest evidence has suggested that ILC2s have the highest level of AhR expression among all ILCs in the gut and that the AhR suppresses the ILC2 transcription program (23). Meanwhile, the AhR sustains ILC3s to control the ILC2-ILC3 balance (23). Additionally, a study of ILCs using AhR knockout (KO) mice indicated that a distinct ILC1/NK cell subtype in the liver, characterized as CD49a^+^TRAIL^+^CXCR6^+^DX5^−^NK1.1^+^, failed to perform its memory and cytotoxic functions (24). In this study, the authors also proposed that environmentally-derived AhR ligands initially drive systematic immunity changes and subsequently affect liver-resident ILC1s (24).

Although the issue of whether exogenous AhR ligands are indispensable in innate immunity is controversial and obscure (22, 23), a recent study has provided compelling data to support that food-derived AhR ligands are essential in mediating innate immune responses. A diet with sufficient plant-derived AhR ligands has been shown to trigger AhR signaling, sustain the ILC3 subset, and further direct the formation of cryptopatches and ILFs in the neonatal gastrointestinal (GI) tract of mice (18). Moreover, in adult mice, a diet abundant in AhR ligands may transiently contribute to the expansion of ILC3s (18). Cruciferous vegetables, which naturally contain potent genotoxic compounds, for example, 1-methoxy-3-indolylmethyl alcohol (1-MIM-OH), I3C, ICZ, etc., trigger ILC3s to produce large amounts of IL-22 (19). Subsequently, IL-22 produced by ILC3s eliminates the transformed intestinal stem cells by the DNA damage response (DDR) and protects the stem cell niche and barrier integrity (19). This finding supports the hypothesis that some extracts of cruciferous vegetables structurally resemble dioxin, so that they may have similar biological activity to this environmental hormone after binding to the AhR (38). Strikingly, a study has demonstrated that AhR signaling needs to be precisely controlled, as excessive AhR activation could actually lead to the loss of ILC3s (20). AhR activation promotes induction of cytochrome P450 1 (CYP1) enzymes, which in turn oxygenate dietary AhR ligands and further diminish their toxicity. As such, the excessive induction of CYP1 enzymes severely depletes natural AhR ligands, causing the reduction of ILC3s and IL-22 in the GI tract. In contrast, supplementary oral intake of natural AhR ligands reverses such effects on IL-22-producing ILC3s, which indicates that dietary AhR ligands are a major contributor to AhR induction and its feedback (20).

## Vitamin A Metabolites, Retinoic Acid (RA) and *All-Trans* RA (atRA), Regulate Developmental Pathways and Migration of ILC subsets

Vitamin A, a fat-soluble vitamin, is enriched in vegetables like squash, sweet potatoes and carrots, fruits such as papaya and nectarine, dairy products, beef and lamb livers, several sea fishes, and so on. Vitamin A deficiency is a global public health concern, particularly in children, leading to poor health conditions (46). Importantly, mammals cannot synthesize vitamin A but can only obtain it from food sources.

Of note, vitamin A bioactive metabolites are the regulators of the immune system, because our body cannot directly utilize vitamin A. One active metabolite of vitamin A, RA, not only enhances the visual process and neurogenesis, but also drastically impacts immune responses and has great therapeutic potential in autoimmune diseases (47–50). Migratory dendritic cells (DCs) convert vitamin A to RA in the mouse epithelial tissues, including the intestine, skin, lung, as well as in the associated draining lymph nodes (51). It has also been suggested that sufficient vitamin A intake is essential for regulating adaptive immunity as RA triggers Th cells to react to mucosal inflammation via RA receptor alpha (RARα) (50).

In addition, the interplay between RA and ILCs has recently become clearer. Evidence regarding how RA interacts with ILC3s emerged first. Mielke et al. found that intestinal NCR^+^ ILC3s express RAR encoding *Rara* and *Rarg *genes, and RA promoted IL-22-producing NCR^+^ ILC3s both in the steady state and under colitis conditions in mice (52) (**Figure 1**). Likewise, atRA sustains ILC3s and IL-22 production and protects the intestinal epithelium from invasion by pathogens via the expression of the atRA-dependent transcription factor Hypermethylated in cancer 1 (HIC1) (27). Several studies demonstrate RA’s critical role in controlling ILC3s in both antenatal and postnatal stages. van de Pavert et al. have revealed that sufficient dietary retinoids are essential for the development of LTi cells in the fetus, and for LTi-dependent embryonic lymphoid organ formation (26). Also, a vitamin A-deprived diet hinders the proliferation of ILC3s and the maturation of secondary lymphoid tissues (53). RA signaling not only affects ILC3s but also ILC2s. Indeed, it has been reported that mice fed a vitamin A-deprived diet suffered from profoundly diminished ILC3s and reduced IL-22 and IL-17 in the gut, meanwhile ILC2s and their corresponding cytokines such as IL-13, IL-4, and IL-5 were elevated (25). A vitamin A-deprived diet leads to IL-13-producing ILC2 expansion under helminth infection to eliminate the worms, probably via increased acquisition and utilization of fatty acids (25, 29). On the other hand, exogenous delivery of RA resulted in a dramatic accumulation of ILC3s, whereas it impaired the maturation of ILC2s (25). Absence of vitamin A or impaired RA signaling resulted in changes in the gene expression profile of ILC2s, e.g., decreased expression of the hexokinase-encoding gene *hk2* and upregulated expression of Peroxisome proliferator-activated receptor alpha (*Ppara*), and ILC2s alternatively uptake more fatty acids to survive and resolve helminth infection (29). These observations suggest that RA differentially promotes ILC subsets rather than universally enhancing ILCs. Hence, vitamin A metabolites are flexible and influential mediators in innate immunity.

A couple of years ago, Wang and coworkers reported a novel and distinct IL-10-expressing-ILC subgroup, regulatory ILC (ILCreg), in both mouse and human intestines (54). In this study, the authors also showed that ILCreg represses the activated ILC1s and ILC3s that are driven by the inflammation in the gut (54). On the other hand, Bando and colleagues recently indicated that there was no clear evidence supporting the universal existence of ILCreg in murine small and large intestines, neither in the steady state nor under experimental colitis conditions (55). Instead, ILC2s might be the predominant source of the anti-inflammatory cytokine IL-10, although IL-10 expression on ILC2s could be induced by IL-2, IL-4, IL-27, and IL-10 itself *in vitro* (55). Indeed, evidence showed that RA, together with IL-2 and IL-33, could stimulate some airway ILC2s to transform into IL-10-producing ILC2s, termed ILC2_10_s, *in vitro* (56). In accordance with this finding, another *in vitro* experiment verified that RA expressed by epithelial cells could switch human ILC2s to IL-10-producing ILC2s (named ILCregs in this report), in inflamed tissue in the respiratory tract but not the tissues in healthy subjects (30). Therefore, RA has an influential role in mediating ILC development and functions during the inflammatory state. Notably, this study also pointed out that airway ILCregs are genetically distinct from intestinal ILCregs due to the key ILC2 markers they express, i.e., GATA3 and ST2, but not Id3 (30). The crosstalk between vitamin A and ILC2s has also been assessed in the pancreatic islets of diabetic mice. IL-10-producing ILC2s, rather than conventional ILC2s, are mainly responsible for protecting the pancreatic islets (57). Indeed, pancreatic islet mesenchymal-derived IL-33 stimulates ILC2s to secrete IL-13 and colony-stimulating factor 2, which in turn elicit pancreatic DCs and macrophages to convert vitamin A to RA, ultimately enhancing insulin secretion by islet β cells (31). Thus, proper interactions between RA and ILC2s could be beneficial in maintaining homeostasis in multiple organs including the lung, intestine and pancreas.

The mechanisms of ILC migration have only recently been characterized. It has been well characterized that chemokine (C-C motif) ligand 25 (CCL25) binds to C-C motif chemokine receptor 9 (CCR9), which is highly expressed by the GI tissues, to mediate gut homing of various immune cells (58). In addition, lymphocyte integrin α4β7 has also been shown to selectively adhere to the mucosal vascular addressin MAdCAM-1, which is expressed by mucosal venules and directs lymphocyte traffic to the gut mucosa (59). Indeed, a recent study found that RA induces the expression of several gut homing receptors, including CCR9 and α4β7, on ILC1s and ILC3s respectively (28), upon exposure of peripheral ILCs to RA. Both CCR9 and α4β7 guide ILC1s and ILC3s to migrate to the gut (28). On the other hand, ILC2s did not undergo this program. ILC2 precursors in the bone marrow intrinsically express CCR9, which dictates their migration to the intestine without RA signaling (28). Collectively, RA acts as a powerful mediator that uses diverse and sophisticated programs to regulate ILC-migration and ILC-related immune responses (**Table 1** and **Figure 1**).

## The Vitamin D Signaling Pathway Modulates ILC3 and Alleviates Intestinal Inflammation

Vitamin D is a fat-soluble vitamin that is mainly produced endogenously when ultraviolet rays from sunlight strike the skin and trigger vitamin D synthesis. Vitamin D is also naturally present in some foods, such as red meat, ocean fish, and eggs, and in fortified milk. It plays an important role in regulating calcium absorption and facilitating normal immune functions (60).

The active forms of vitamin D, including 1α, 25-Dihydroxyvitamin D3 (1,25D3) and 1,25-Dihydroxyvitamin D (1,25D), together with atRA may control Th17 and regulatory T cell (Treg) development and suppress multiple experimental autoimmune diseases in the gut, brain, and skin (61–63). Vitamin D and vitamin D receptor (VDR) are potent immunoregulatory factors involved in adaptive and innate immunity and dysbiosis (37, 64). It has already been reported that immune cells such as Th17 cells, DCs and macrophages express a high level of VDR (65). Furthermore, emerging evidence indicates that enteric ILC3s, which express the IL-23R, are a significant target for vitamin D signaling in immune responses, as VDR expression by ILC3s is even higher than by Th17 cells (34). Konya and colleagues have further verified that human ILC3s activated by IL-23 and IL-1β respond vigorously to 1,25D, upregulating numerous VDR-associated genes (36). Meanwhile, 1,25D suppresses the IL-23R pathway and IL-23-associated cytokines such as IL-22, IL-17F in ILC3s (36). Similarly, in endogenous vitamin D ligand-deficient (Cyp27B1 KO) mice, a vitamin D sufficient diet could recover the impaired vitamin D signaling pathway, promote IL-22 secreting ILC3s, and prevent acute enteric inflammation. Conversely, a vitamin D deficient diet fails to do so (35). In agreement with these basic studies, vitamin D deficiency is also an independent risk factor for inflammatory bowel diseases (IBD) (36, 66). Several clinical trials also suggest that vitamin D is beneficial in patients with IBD by regulating the composition of bacteria in the gut (67, 68).

Despite these insights, the crosstalk between vitamin D, VDR, and ILCs is complicated and not yet fully understood. A study that used VDR KO mice indicated that the deletion of VDR enhanced IL-22-producing ILC3s in the small intestine (37). It subsequently reshaped the gut microbiome, and the VDR KO mice were less susceptible to *Citrobacter rodentium (C. rodentium)* (37). Although still controversial, He et al. reported that vitamin D/VDR signaling controlled the development and proliferation of ILC3s, mainly LTi-like ILC3s in the gut in a commensal bacteria-independent manner and dramatically enhanced host defense against *C. rodentium* (34) (**Table 1**). Further study is needed to clarify the mechanisms of how vitamin D regulates ILCs.

## Dietary Nutrients Activate NK Cells and Promote Immunotherapy Against Cancer

NK cells, which express surface markers such as NK1.1 and NKp46 in mice and NK1.1, NKp44, CD16, and CD56 in humans, have potent cytotoxic functions and reside within tissues or circulate in the blood (1). Unlike other ILCs, NK cells have long been known for their essential role in mediating immune responses and their anti-cancer properties (69). They also play defense roles against viral, bacterial, and parasitic infections. However, in patients who suffer from cancer, NK-cell activity is often impaired (70, 71).

Recently, a work from Song and collaborators showed that atRA recruits NK cells to infiltrate tumors and exert their cytotoxic function in a melanoma mouse model (32). Likewise, atRA improves the lytic activity of the antitumor agent, anti-CD38 monoclonal antibody, against multiple myeloma cells (33). Furthermore, vitamin D could enhance the function of NK cells. Supplementary vitamin D could act as a stimulator of splenic NK cells in a mouse model but not in obese mice (72).

Herbs have also been found to activate NK cells. Herbs originated in ancient times and have been used since in our daily diets. Curcumin is a major chemical of *Curcuma longa* plants that belongs to the ginger family, and it is a common spice in Asian cuisines like curry powders (73). Several studies have suggested that curcumin is an immunotherapeutic agent against tumors (74, 75). Indeed, curcumin has been reported to recruit activated NK cells to glioblastoma stem cells and eliminate the tumor cells in a mouse model (76). Asian ginseng, one of the most widely used herbs globally, has also been extensively investigated for its antimicrobial and anticancer capabilities (77). One group reported that ginseng extracts rely on the IFN-*γ* pathway to promote NK cell cytotoxic activity, while ginsenoside itself hardly showed any NK cell-promoting ability (78). An *in vitro* analysis established that wild ginseng extracts significantly potentiated NK cell antitumor activity via upregulated IL-2 responsiveness and granzyme B, a cytotoxic protease secreted by NK cells to cause apoptosis of target cells (79).

## Concluding Remarks and Perspectives

The crosstalk between ILCs and food-derived microelements has provided a new perspective on innate immunity; natural compounds acquired from our daily diet control and regulate innate immunity through various mechanisms. Nevertheless, several fundamental issues have not yet been addressed.

As we discussed earlier, it is apparent that the sources of AhR ligands are still under debate. Despite the anti-inflammatory actions of dietary AhR ligands, we also have to consider the contradictory observations of AhR ligands in tumorigenesis; AhR ligands have both pro-cancer and anti-cancer effects (15). Thus, their therapeutic efficacy needs to be explored more thoroughly.

To note, intestinal ILCreg’s existence is controversial, and it has been proposed that intestinal ILCregs might be a subset of ILC2s rather than an independent cell type distinct from ILCs or Tregs (30, 55). And further investigation is required to clarify the function and differentiation of ILCreg in the gut.

Striking evidence has shown that vitamins are essential micronutrients to maintain health, and more importantly, they serve as important immunoregulators. From what we have discussed above, it clearly suggests that supplementary vitamin A could be beneficial for mucosal tissue integrity under inflammation stress, especially for young children, although further clinical trials are needed to confirm its effects. Although evidence suggested that an adequate level of vitamin D in the bloodstream may lower the risk of colorectal cancer (67, 80), and vitamin D plays critical roles in innate immunity, its functions are still controversial.

Compelling data have indicated that herbs enhance NK cell actions and are beneficial in cancer treatment. Nonetheless, studies focused on other ILCs and herbs are lacking. Besides, most studies regarding NK cells and herbs are restricted to *in vitro* assessment. However, an herb Daikenchuto, which is composed of Asian ginseng, pepper and processed ginger, has been intensively investigated for its anti-inflammatory effects and colonic transit activity (81, 82). Daikenchuto could be a promising drug to overcome GI inflammation and gut dysmotility and deserves more in-depth investigation. Therefore, a more comprehensive understanding of the molecular effects of herbs on ILCs is in high demand.

In conclusion, food-derived micronutrients are indispensable to innate immunity and protect the mucosal barrier from damage and maintain the host microenvironment. Further studies are needed to validate a wider variety of micronutrient effects on promoting human health and understanding disease pathogenesis, which may eventually provide insight for therapies in immune-related diseases and contribute to drug development.

## Author Contributions

ZZS and NS-T wrote manuscript. HO provided critical comments and finalized the manuscript. All authors contributed to the article and approved the submitted version.

## Funding

NS-T was supported by the Japan Society for the Promotion of Science KAKENHI (18K07189) and Yakult Bio-Science foundation. HO was supported by the Japan Society for the Promotion of Science KAKENHI (19H01030 and 19F19785).

## Conflict of Interest

The authors declare that the research was conducted in the absence of any commercial or financial relationships that could be construed as a potential conflict of interest.
